# Biphasic Effect of Diabetes on Neuronal Nitric Oxide Release in Rat Mesenteric Arteries

**DOI:** 10.1371/journal.pone.0156793

**Published:** 2016-06-07

**Authors:** Esther Sastre, Laura Caracuel, Javier Blanco-Rivero, María Callejo, Fabiano E. Xavier, Gloria Balfagón

**Affiliations:** 1 Departamento de Fisiología, Facultad de Medicina, Universidad Autónoma de Madrid, Madrid, Spain; 2 Instituto de Investigación La Paz (IdIPAZ), Madrid, Spain; 3 Departamento de Fisiologia e Farmacologia, Universidade Federal de Pernambuco, Recife, Brazil; Max-Delbrück Center for Molecular Medicine (MDC), GERMANY

## Abstract

**Introduction:**

We analysed possible time-dependent changes in nitrergic perivascular innervation function from diabetic rats and mechanisms implicated.

**Materials and Methods:**

In endothelium-denuded mesenteric arteries from control and four- (4W) and eight-week (8W) streptozotocin-induced diabetic rats the vasoconstriction to EFS (electrical field stimulation) was analysed before and after preincubation with L-NAME. Neuronal NO release was analysed in the absence and presence of L-arginine, tetrahydrobiopterine (BH4) and L-arginine plus BH4. Superoxide anion (O_2_^-^), peroxynitrite (ONOO^-^) and superoxide dismutase (SOD) activity were measured. Expressions of Cu-Zn SOD, nNOS, p-nNOS Ser^1417^, p-nNOS Ser^847^, and Arginase (Arg) I and II were analysed.

**Results:**

EFS response was enhanced at 4W, and to a lesser extent at 8W. L-NAME increased EFS response in control rats and at 8W, but not at 4W. NO release was decreased at 4W and restored at 8W. L-arginine or BH4 increased NO release at 4W, but not 8W. SOD activity and O_2_^-^ generation were increased at both 4W and 8W. ONOO^-^ decreased at 4W while increased at 8W. Cu-Zn SOD, nNOS and p-NOS Ser^1417^ expressions remained unmodified at 4W and 8W, whereas p-nNOS Ser^847^ was increased at 4W. ArgI was overexpressed at 4W, remaining unmodified at 8W. ArgII expression was similar in all groups.

**Conclusions:**

Our results show a time-dependent effect of diabetes on neuronal NO release. At 4W, diabetes induced increased O_2_^-^ generation, nNOS uncoupling and overexpression of ArgI and p-nNOS Ser^847^, resulting in decreased NO release. At 8W, NO release was restored, involving normalisation of ArgI and p-nNOS Ser^847^ expressions.

## Introduction

Cardiovascular disorders including hypertension, atherosclerosis and ischemic heart and cerebral diseases are the most common cause of morbidity and mortality in diabetic patients [1]. Altered blood vessel function is the common factor among these cardiovascular complications; it represents a problem of great clinical importance underlying the development of various severe complications and may occur even in the early stages of diabetes in both large and small vessels [2, 3]. Although the precise mechanism behind diabetes-associated vascular disorders remains incompletely understood, in most cases it involves changes in the production or bioavailability of nitric oxide (NO) [3].

NO is a biomolecule that plays a critical role in neurotransmission, vascular homeostasis, immune response, etc. In the vascular wall, NO is mainly produced by endothelial cells and nitrergic neurons [4, 5]. The lack or excess of NO production in the vascular system can present several important pathological implications [4, 6]. In diabetes, alterations of endothelium-derived NO and their implications have been exhaustively studied, but abnormalities in vascular neuronal NO production have been scarcely investigated. It is important to mention that altered NO production in diabetes is not a homogeneous process in its characteristics and distribution, varying with the duration of the exposure to hyperglycemia and the tissue involved [7–9].

In rat mesenteric arteries, NO is released from nitrergic innervation where it is synthesised through nNOS activity [10]. Its synthesis and release is closely linked to the formation of reactive oxygen species [11]. In diabetes, the few results published about nitrergic innervation function are contradictory. The available studies indicate: 1) dysfunction in rat cerebral arteries, corpus cavernosum and gastrointestinal organs [12–14]; 2) normal function in rat urethral smooth muscle [15] and 3) the existence of a biphasic pattern in nitrergic innervation function: one in which the function is preserved, the other in which function is progressively impaired [8].

In diabetes increased oxidative and nitrosative stress triggers several pathways that affect endothelial NO synthesis and metabolism [6]. Decreases in L-arginine and/ or BH4 account for NOS uncoupling leading to the generation of superoxide anions and other ROS [16]. It is well known that, similarly to eNOS and iNOS, the essential co-factor tetrahydrobiopterin (BH4) and the substrate L-arginine play a key role in the mechanism of neuronal NO synthesis [16]. In addition, a deficit of BH4 through oxidation to BH2 and of L-arginine through arginase overexpression has been reported in diabetes [17, 18].

In view of this, the present experiments were designed to study the possible time-dependent changes of nitrergic perivascular innervation function in diabetic rats, with special reference to the role of the redox state.

## Materials and methods

### Ethic statement

The investigation conforms to the European Commission Directive 86/609 CEE Art. 21 (1995), and the Guide for the Care and Use of Laboratory Animals published by the US National Institutes of Health (NIH Publication No. 85–23, revised 1996). This study has been approved by the ethical committee of the Universidad Autónoma de Madrid.

### Animal housing

Three month-old male Sprague-Dawley rats were used in this study. Animals were divided in three experimental groups: 1) Control animals; 2) Four-week diabetic rats and 3) Eight-week diabetic rats. Diabetes was induced by a single intraperitoneal administration of streptozotocin (60 mg/kg) dissolved in citric acid-trisodium citrate (0.1 mol/L, pH = 4.5) as vehicle. The control group was inoculated only with vehicle. To prove the effectiveness of the treatment, glucose measurements were performed in blood samples from the tail tip, using a diagnostic autoanalyser (Optium Xceed, Abott, Spain), and diabetes was considered to be established once fasting glucose values rose above 200 mg/dl.

Body weight was measured at the beginning and end of treatment. Rats were housed at a constant room temperature, humidity and 12 h light/dark cycle and had free access to tap water and standard rat chow. Animals were sacrificed by CO_2_ inhalation; superior mesenteric artery was removed and placed in cold Krebs−Henseleit solution (KHS, in mmol/L: NaCl 115; CaCl_2_ 2.5; KCl 4.6; KH_2_PO_4_ 1.2; MgSO_4_∙7H_2_O 1.2; NaHCO_3_ 25; glucose 11.1, Na_2_ EDTA 0.03) at 4°C.

### Vascular Reactivity Study

The method used for isometric tension recording has been described in full elsewhere [19, 20]. Two parallel stainless steel pins were introduced through the lumen of superior mesenteric artery segments: one was fixed to the bath wall, and the other connected to a force transducer (Grass FTO3C; Quincy, Mass., USA); this, in turn, was connected to a model 7D Grass polygraph. For EFS experiments, segments were mounted between two platinum electrodes 0.5 cm apart and connected to a stimulator (Grass, model S44) modified to supply adequate current strength. Segments were suspended in an organ bath containing 5 mL of KHS at 37°C and continuously bubbled with a 95% O_2_ to 5% CO_2_ mixture (pH of 7.4). Experiments were performed in endothelium-denuded segments to eliminate the main source of vasoactive substances, including endothelial NO. This avoided possible actions of different drugs on endothelial cells that could lead to misinterpretation of results. Endothelium was removed by gently rubbing the luminal surface of the segments with a thin wooden stick. The segments were subjected to a tension of 0.5 g, which was readjusted every 15 min during a 90-min equilibration period before drug administration. After this, the vessels were exposed to 75 mmol/L KCl, to check their functional integrity. Endothelium removal did not alter the contractions elicited by 75 mmol/L KCl. After a washout period, the absence of vascular endothelium was tested by the inability of 10 μmol/L acetylcholine to relax segments precontracted with noradrenaline.

Frequency-response curves to electrical field stimulation (EFS, 1, 2, 4 and 8 Hz) were performed. The parameters used for EFS were 200 mA, 0.3 ms, 1–8 Hz, for 30 s with an interval of 1 min between each stimulus, the time required to recover basal tone. To prove the neuronal origin of EFS-induced contractile response, segments were incubated with nerve impulse blocker tetrodotoxin (TTX, 0.1 μmol/L). A washout period of at least 1 h was necessary to avoid desensitisation between consecutive curves. Two successive frequency-response curves separated by 1-hour intervals produced similar contractile responses.

To analyse the participation of NO in the EFS-induced response in segments from control and diabetic animals, 0.1 mmol/L N^ω^-nitro-L-arginine methyl ester (L-NAME), a non-specific inhibitor of nitric oxide synthase (NOS), was added to the bath 30 min before performing the second frequency–response curve.

### Nitric Oxide Release

NO release was measured using fluorescence emitted by the fluorescent probe 4,5-diaminofluorescein (DAF-2), as previously described [21, 22]. Endothelium-denuded mesenteric arteries from all experimental groups were subjected to a 60-minute equilibration period in HEPES buffer (in mmol/L: NaCl 119; HEPES 20; CaCl_2_ 1.2; KCl 4.6; MgSO_4_ 1; KH_2_PO_4_ 0.4; NaHCO_3_ 5; glucose 5.5; Na_2_HPO_4_ 0.15; pH 7.4) at 37°C. Arteries were incubated with 2 μmol/L DAF-2 for 30 min. The medium was then collected to measure basal NO release. Once the organ bath was refilled, cumulative EFS periods of 30 s at 1, 2, 4 and 8 Hz were applied at 1 min intervals. Afterwards, the medium was collected to measure EFS-induced NO release. The fluorescence of the medium was measured at room temperature using a spectrofluorometer (Optima, BMG Labtech, Ortenberg, Germany) with excitation wavelength set at 492 nm and emission wavelength at 515 nm.

The EFS-induced NO release was calculated by subtracting basal NO release from that evoked by EFS. Also, blank sample measures were collected in the same way from segment-free medium in order to subtract background emission. Some assays were performed in the presence of 0.1 mmol/L 7 nitroindazol (7-NI, a specific nNOS blocker), 0.1 μmol/L TTX, 3 μmol//L L-arginine, 0.1 μmol/L BH4 or with a combination with both L-arginine and BH4. The amount of NO released was expressed as arbitrary units/mg tissue.

### Detection of superoxide anions

Superoxide anion levels were measured using lucigenin chemiluminescence, as previously described [22]. Endothelium-denuded mesenteric segments from all experimental groups were rinsed in KHS for 30 min, equilibrated for 30 min in HEPES buffer at 37°C, transferred to test tubes that contained 1 mL HEPES buffer (pH 7.4) containing lucigenin (5 μmol/L) and then kept at 37°C. The luminometer (Optocom I, GEM Biomedical Inc., Hamden, U.S.A.) was set to report arbitrary units of emitted light; repeated measurements were collected for 5 min at 10 s intervals and averaged. 4,5-Dihydroxy-1,3-benzene-disulphonic acid ‘‘tiron” (10 mmol/L), a cell-permeant, non-enzymatic superoxide anion scavenger, was added to quench the superoxide anion-dependent chemiluminescence. Calculations were performed subtracting the lucigenin chemiluminescence obtained in the presence of tiron from that obtained in its absence. Blank measures were collected in the same way without mesenteric segments to subtract background emission.

### Peroxynitrite detection

Peroxynitrite levels were measured using fluorescence emitted by the fluorescent probe dihydrorhodamine 123 (DHR), as previously described [23]. Endothelium-denuded mesenteric arteries from all experimental groups were subjected to a 30-minute equilibration period in Phosphate-buffered-saline solution (PBS, in mmol/L: NaCl 137; KCl 2.7; Na_2_HPO_4_.2H_2_O 10; KH_2_PO_4_ 2; pH 7.4) at 37°C. Arteries were incubated with 5 μmol/L DHR for 30 min. The medium was then collected to measure basal peroxynitrite release. Once the organ bath was refilled, cumulative EFS periods of 30 s at 1, 2, 4 and 8 Hz were applied at 1 min intervals. Afterwards, the medium was collected to measure EFS-induced peroxynitrite release. The fluorescence of the medium was measured at room temperature using a spectrofluorometer (Optima, BMG Labtech, Ortenberg, Germany) with excitation wavelength set at 500 nm and emission wavelength at 536 nm. The EFS-induced ONOO^-^ release was calculated by subtracting basal NO release from that evoked by EFS. Blank sample measures were collected in the same way from segment-free medium in order to subtract background emission. The amount of ONOO^-^ released was expressed as arbitrary units/mg tissue.

### Western Blot

Western blot analysis of Cu-Zn superoxide dismutase, nNOS and phosphorylated nNOS, and arginase 1 and 2 expressions were performed as previously described [24]. For these experiments, we used rabbit monoclonal SOD 1 antibody (1:10000, Abcam), mouse monoclonal nNOS antibody (1:1000, BD Transduction Laboratories), rabbit polyclonal p-nNOS Ser^1417^ and p-nNOS Ser^847^ antibodies (1:2000, Abcam), mouse monoclonal arginase 1 (1:2000, BD Transduction Laboratories) and rabbit polyclonal arginase 2 (1:1000, santa Cruz Biothecnology, Inc).

### Superoxide Dismutase activity

Frozen samples of mesenteric segments from all experimental groups were homogenised in ice cold 0.1 mmol/L Tris/HCl, pH 7.4 containing 0.5% Triton X-100, 5 mmol/L β-mercaptoethanol and 0.1mg/mL PMSF. After centrifugation at 14000 g (5 min, 4°C) 20 μl of supernatants were used in the assay. Enzyme activity was measured following the manufacturer’s instructions with a colorimetric superoxide dismutase activity assay kit (Colorimetric) (Abcam). The superoxide dismutase activity was expressed as arbitrary units/ μg protein.

### Drugs used

L-noradrenaline hydrochloride, acetylcholine chloride, lucigenin, Tiron, DAF-2, dihydrorhodamine, L-NAME, 7-NI, TTX, L-arginine and BH4 (Sigma-Aldrich, Madrid, Spain) were used. Stock solutions (10 mmol/L) of most drugs were made in distilled water; noradrenaline was dissolved in a NaCl (0.9%)-ascorbic acid (0.01% w/v) solution and DAF-2, dihydrorhodamine and 7-NI were dissolved in dimethyl sulfoxide. These solutions were kept at –20°C and appropriate dilutions were made in KHS on the day of the experiment.

### Data Analysis

The responses elicited by EFS were expressed as a percentage of the initial contraction elicited by 75 mmol/L KCl for comparison between arteries from control and diabetic animals. Results are given as mean ± SEM. Statistical analysis was done by comparing the curve obtained in the presence of the different substances with the previous curve by means of repeated measure analysis of variance (ANOVA) followed by Bonferroni post-hoc test, using GraphPad Prism 5.0 software (CA, USA). For NO, superoxide anion and peroxynitrite release data, Western blot and SOD activity assays, statistical analysis was done using one-way ANOVA followed by Newman-Keuls *post-hoc* test for unpaired experiments. P<0.05 was considered significant.

## Results

### Animal evolution

Blood glucose concentration was higher in four-week and eight-week diabetic rats, compared to control rats 72 h after streptozotocin or vehicle inoculation. Initial body weight was similar in animals from all groups. Final body weight was lower in four-week and eight-week diabetic rats, compared to control rats (Table 1).

**Table 1:** Animal evolution: Analysis of initial and final body weight, and blood glucose levels 72 h. after treatment inoculation in four (4W) and eight (8W) weeks’ diabetic rats.

	Control	4W	8W
**Initial body weight (g)**	301.4 ± 3.6	306.1 ± 5.5	294.5 ± 6.9
**Final body weight (g)**	412.0 ± 12.8	257.8 ± 7.3*	245.1 ± 12.6*
**Blood glucose (mg/dL)**	91.2 ± 23.5	398.8 ± 49.2*	365.6 ± 36.1*

All data are expressed as mean ± S.E.M. n = 12–16 rats each group.

* P< 0.05 vs control.

### Vascular responses to EFS

**Fig 1:**
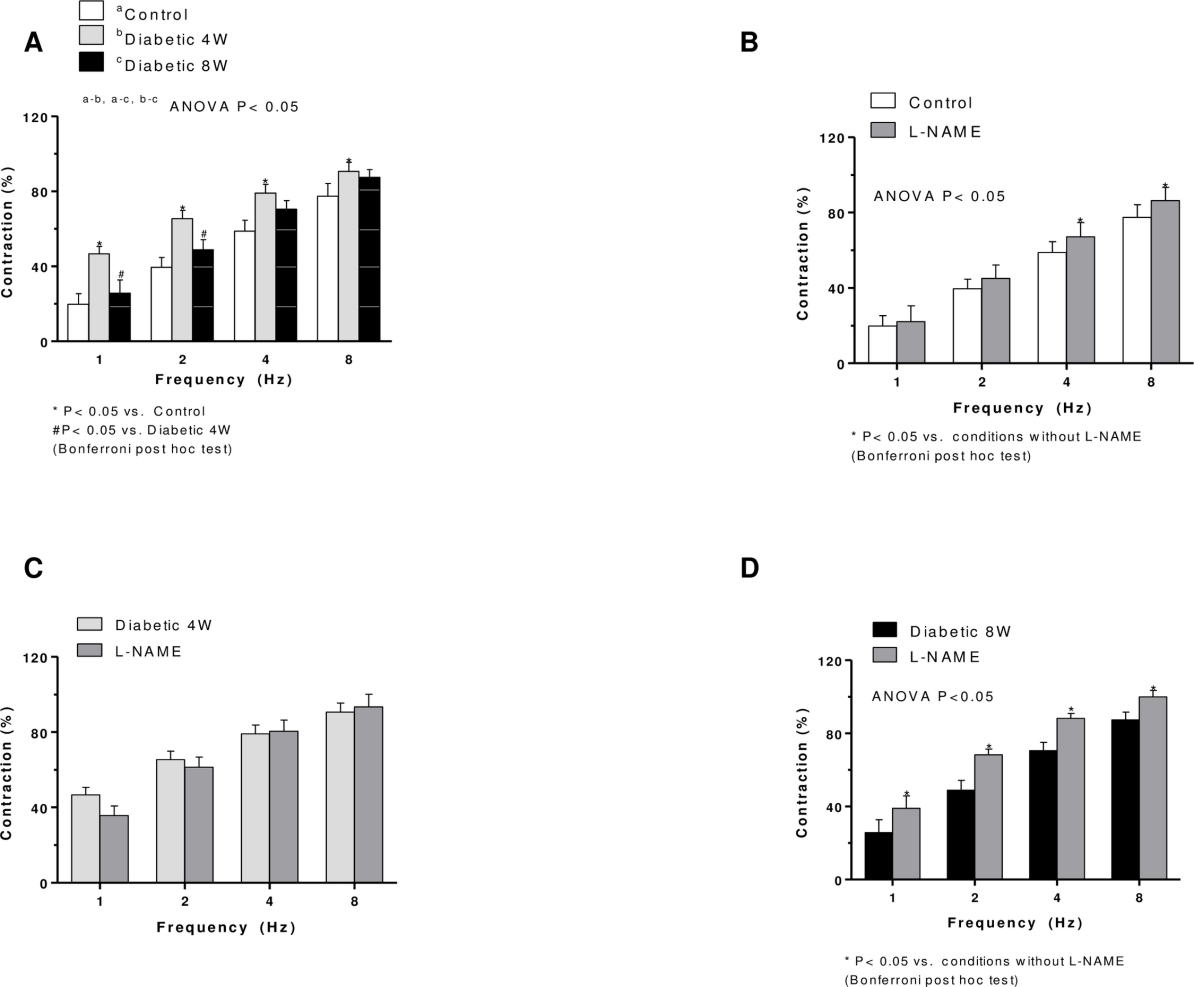
(A). EFS-induced vasoconstriction in endothelium-denuded mesenteric segments from four- (4W) and eight-week (8W) diabetic and non-diabetic rats. Effect of L-NAME on the vasoconstrictor responses induced by EFS in mesenteric segments from control (B), four- (C) and eight-week diabetic (D) rats. Results (mean ± SEM) are expressed as a percentage of the initial contraction elicited by KCl. N = 5–7 animals per group.

EFS produced frequency-dependent contractions (1, 2, 4, and 8 Hz) in mesenteric arteries without endothelium. These contractile responses were greater in segments from four-week diabetic arteries at 1, 2 and 4 Hz than non-diabetic arteries (Fig 1A). In segments from eight-week diabetic rats, EFS-induced contraction was decreased in respect to four-week diabetic rats, but increased in respect to control (Fig 1A). EFS-induced contractions were practically abolished by 0.1 μmol/L TTX in segments from all experimental groups (results not shown). Responses elicited by 75 mmol/L K^+^ were comparable in all groups (results not shown).

Pre-incubation with non-specific or specific NOS inhibitor, L-NAME, increased the EFS-induced vasoconstriction in segments from the non-diabetic and eight-week diabetic rats, but not in the four-week diabetic rats (Fig 1B, 1C and 1D). In segments from eight-week diabetic rats, the L-NAME effect was greater than in non-diabetic rats.

### Nitric oxide release

**Fig 2:**
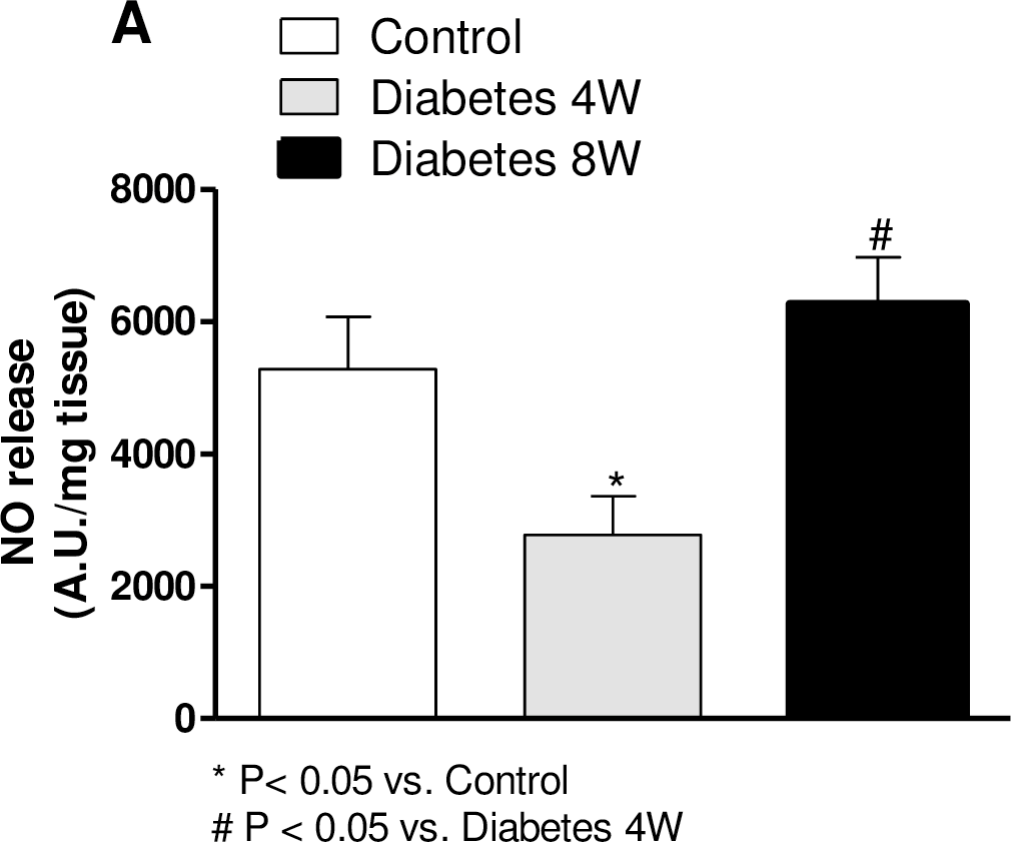
Effect of four (4W) or eight weeks’ (8W) diabetes on EFS-induced NO release in rat mesenteric arteries. Results (mean ± SEM) are expressed as arbitrary (A.U.)·mg^−1^ tissue. One way ANOVA, *P<0.05 *vs*. control, #P<0.05 *vs*. N = 5–7 animals per group.

EFS induced NO release in segments from all groups (Fig 2). This release was decreased in four-week diabetic arteries and remained unmodified in arteries from eight-week diabetic compared to non-diabetic rats (Fig 2). TTX or 7-NI practically abolished EFS-induced NO release in all experimental groups (Table 2).

**Table 2:** Effect of preincubation with 7-nitroindazol (7-NI, 0.1 mmol/L), or 0.1 μmol/L TTX on EFS-induced NO release in mesenteric segments from control, four (4W) and eight (8W) weeks’ diabetic rats.

	Control	4W	8W
**Without inhibitor**	5286 ± 791.5	2778 ± 586.7*	6267 ± 706.7^#^
**+ 7-NI**	94 + 17^+^	86 ± 12^+^	90 ± 17^+^
**+ TTX**	92 ± 13^+^	91 ± 15^+^	83 ± 21^+^

Results (means ± S.E.M.) are expressed in arbitrary units (A.U.)/mg tissue. n = 6–10 animals each group.

* P < 0.05 vs. control animals.

# P < 0.05 8W vs. 4W diabetes- + P< 0.05 vs. conditions without specific inhibitor.

**Fig 3:**
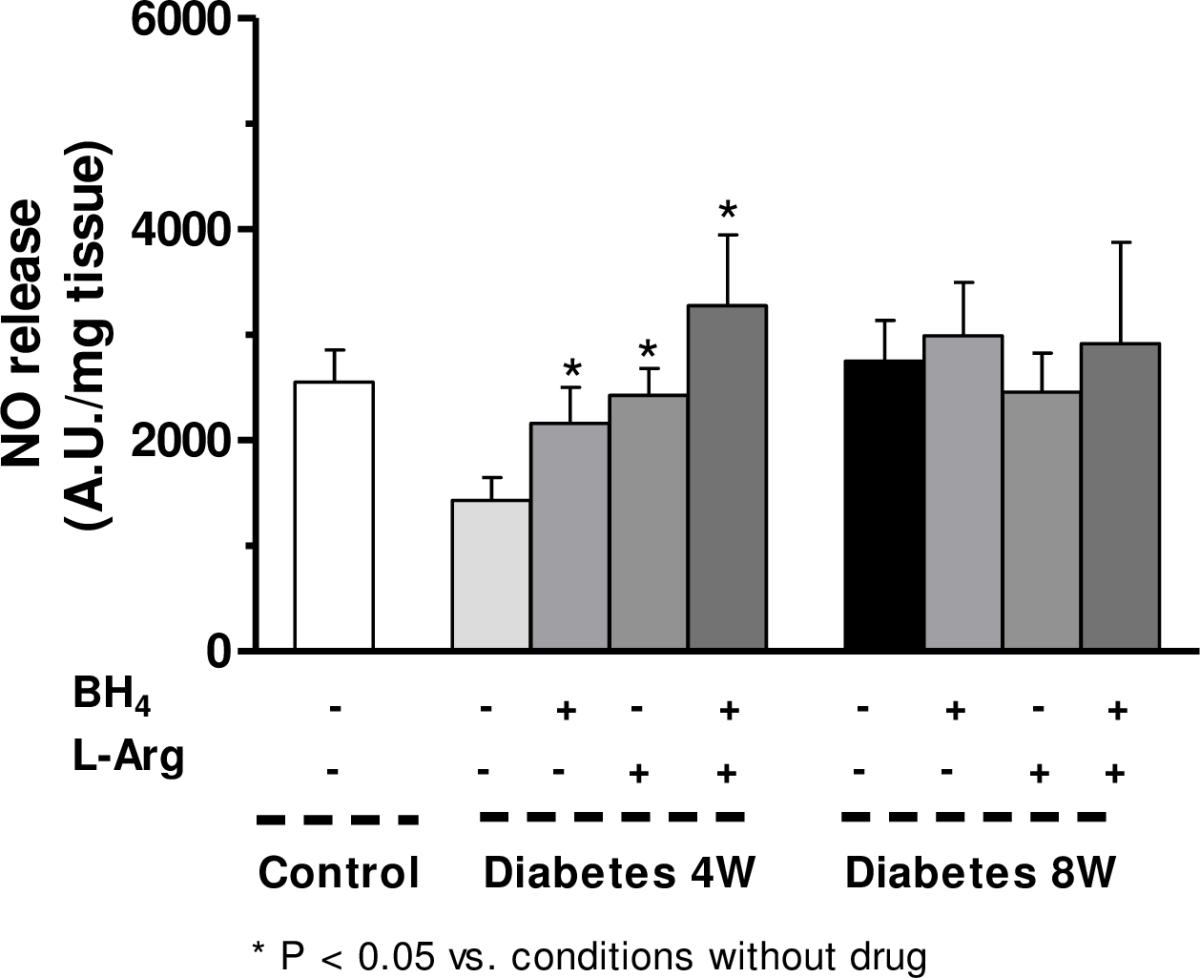
Effect of preincubation with L-arginine, BH4 or L-arginine plus BH4 on the EFS-induced nitric oxide release in mesenteric segments from four- (4W) and eight-week (8W) diabetic rats. Results (mean ± SEM) are expressed as arbitrary (A.U.)·mg^−1^ tissue. Two way ANOVA, *P<0.05 *vs*. without L-arginine or BH4.

Incubation with L-arginine or BH4 restored NO release in segments from four-week diabetic rats (Fig 3). In this group, co-incubation with L-arginine plus BH4 induced an additional increase in NO release compared to L-arginine or BH4 given alone (Fig 3). In eight-week diabetic arteries, L-arginine or BH4 failed to produce any effect on NO release (Fig 3).

### Reactive Oxygen Species Generation

**Fig 4:**
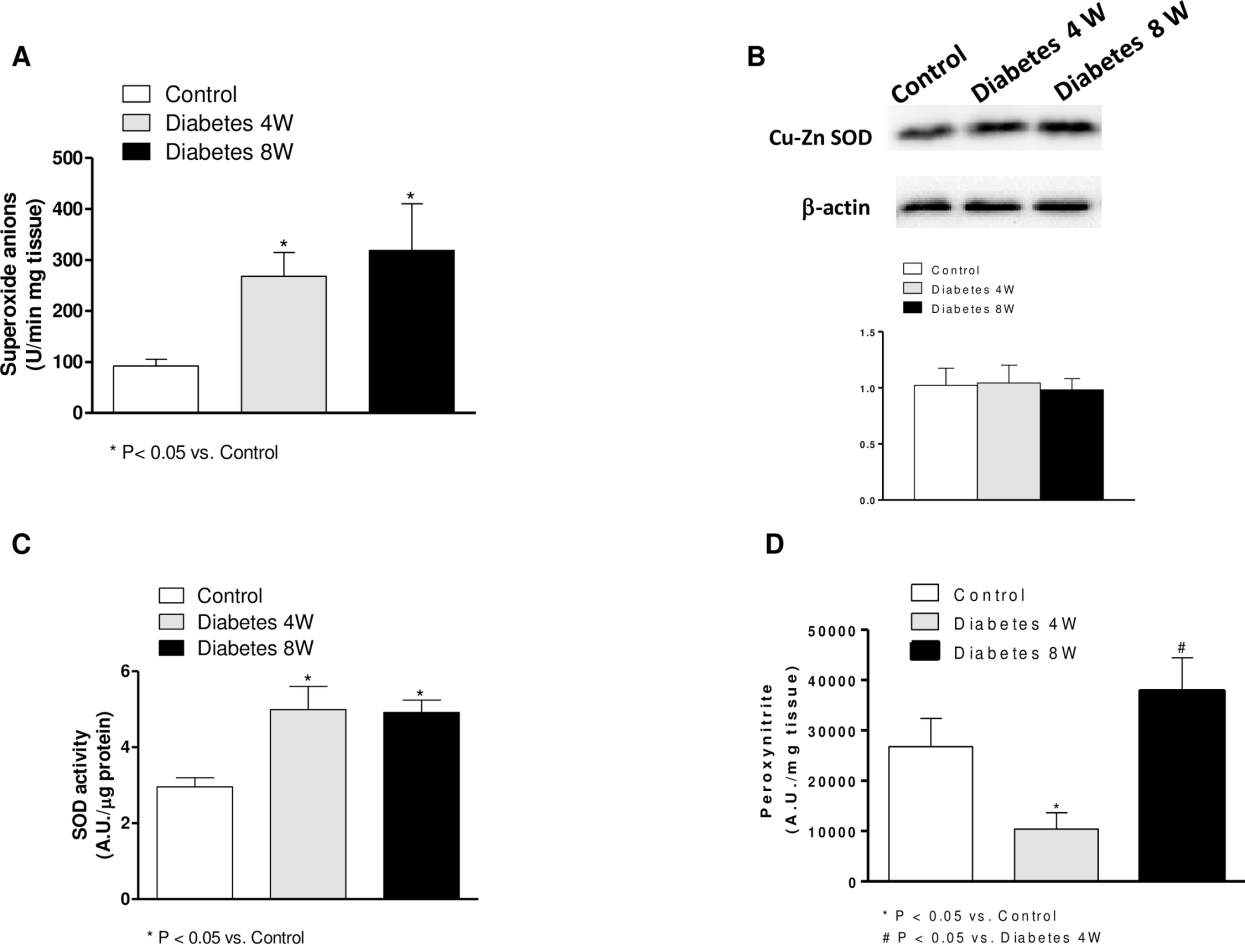
(A). Superoxide anion release in mesenteric segments from control and four- (4W) and eight-week (8W) diabetic rats. Results (mean ± S.E.M.) are expressed as chemiluminiscence units (U)/min mg tissue. (B). Effect of four and eight weeks’ diabetes on peroxynitrite formation in rat mesenteric arteries. Results (mean ± S.E.M.) are expressed as arbitrary units (A.U)/min mg tissue. N = 5–7 animals each group. (C). Superoxide dismutase activity in mesenteric arteries from control and four- and eight-week diabetic rats. Results (mean ± S.E.M.) are expressed as arbitrary units (A.U)/min μg tissue. (D). Effect of four and eight weeks’ diabetes on Cu-Zn superoxide dismutase (SOD) expression. Lower panel shows relation between Cu-Zn SOD expression and β-actin. Results (mean ± S.E.M.) are expressed as ratio of the signal obtained for each protein and the signal obtained for β-actin.

Superoxide anion generation was increased in segments from both four- and eight-week diabetic compared to non-diabetic rats (Fig 4A). ONOO^-^ formation was decreased in four-week diabetic rats while it was increased in segments from eight-week diabetic rats with respect to control rats (Fig 4B)

### Superoxide dismutase activity

Superoxide dismutase activity was increased in mesenteric arteries from both four- and eight-week diabetic than non-diabetic rats (Fig 4C).

### Cu-Zn superoxide dismutase, nNOS, p-nNOS and arginase expressions

**Fig 5:**
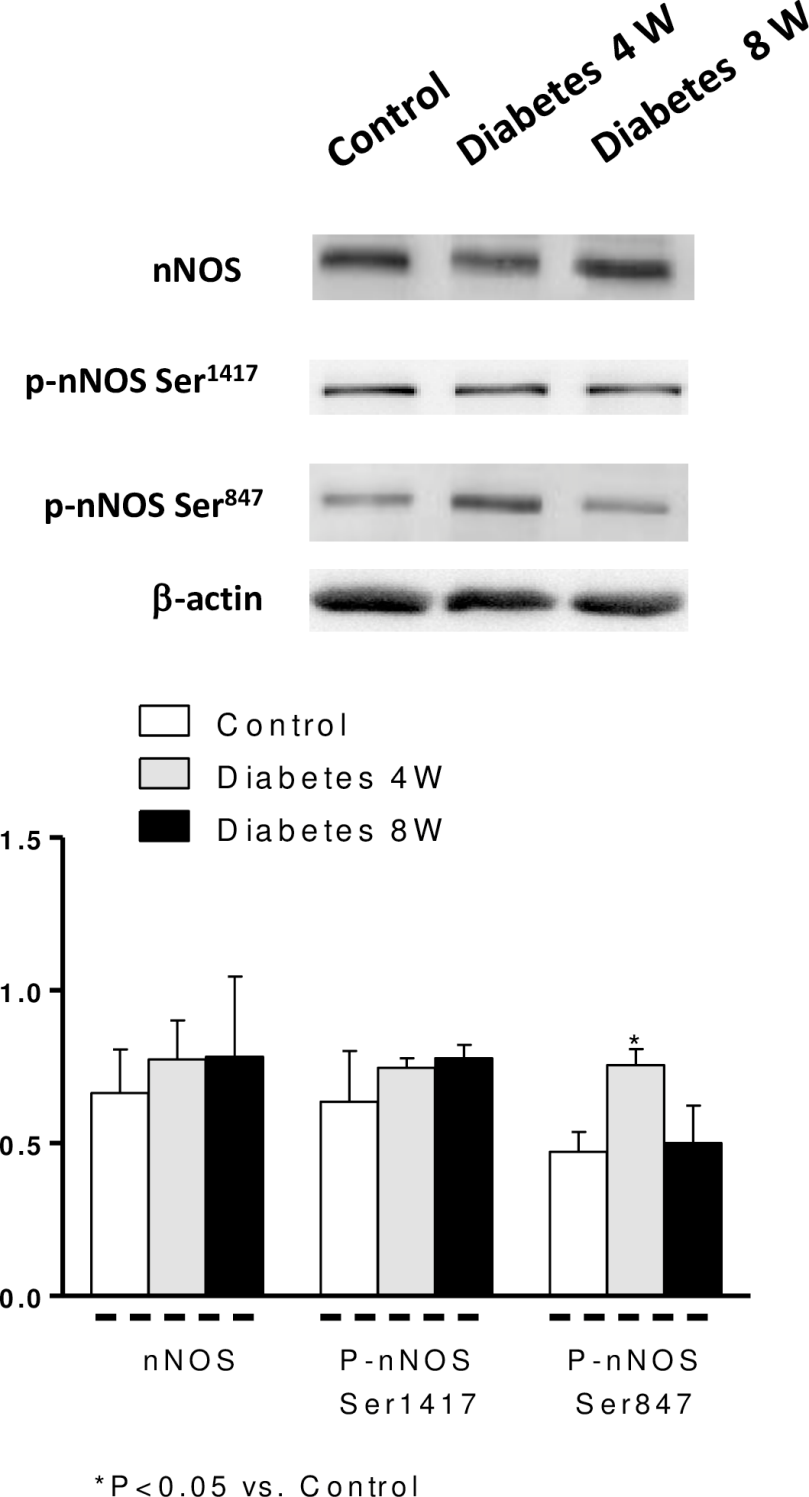
nNOS, p-nNOS Ser^1417^ and p-nNOS Ser^847^ in mesenteric arteries from control and four- (4W) and eight-week (8W) diabetic rats. Lower panel shows relation between nNOS, p-nNOS Ser^1417^ and p-nNOS Ser^847^ expression and β-actin. Results (mean ± S.E.M.) are expressed as ratio of the signal obtained for each protein and the signal obtained for β-actin.

The expressions of Cu-Zn superoxide dismutase (Fig 4D), nNOS and p-NOS Ser^1417^ (Fig 5) were not modified by four or eight weeks’ diabetes, whereas p-nNOS Ser^847^ expression was increased in homogenates from four-week diabetic arteries compared with eight-week diabetic and control segments (Fig 5).

**Fig 6:**
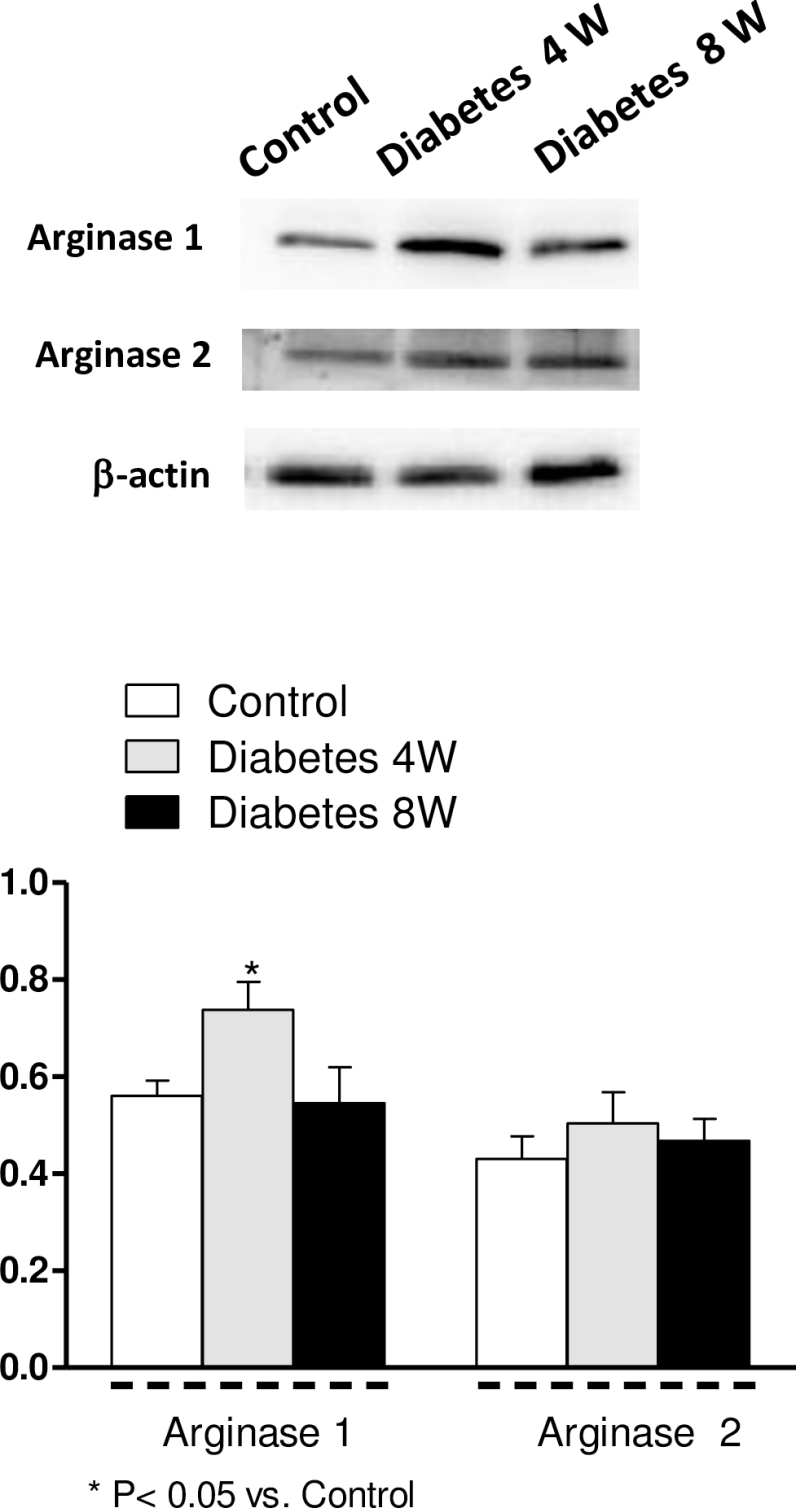
Effect of four (4W) and eight (8W) weeks’ diabetes on arginase I and II expressions. Lower panel shows relation between arginase I or II expression and β-actin. Results (mean ± S.E.M.) are expressed as ratio of the signal obtained for each protein and the signal obtained for β-actin.

Arginase I and II were expressed in mesenteric arteries from all experimental groups (Fig 6). Arginase I was overexpressed in arteries from four-week diabetic rats, but remained unmodified in eight-week diabetic arteries with respect to control values (Fig 6). Expression of arginase II was similar in all groups (Fig 6).

## Discussion

The present study shows time-dependent changes in EFS-induced neuronal NO release in superior mesenteric artery from diabetic rats. In the early stage of diabetes (four weeks from induction), there was a reduction in neuronal NO release that was associated to nNOS uncoupling, increased superoxide anion generation and overexpression of arginase I and nNOS phosphorylation at Ser^847^ (p-NOS Ser^847^). At eight weeks and despite the maintenance of increased oxidative stress, nNOS uncoupling seems to be reversed and arginase I and p-NOS Ser^847^ expression were normalized, restoring NO release. Taken together, these results indicate the existence of different time-dependent mechanisms that regulate perivascular neuronal NO synthesis in mesenteric diabetic arteries.

In all experimental groups, EFS-induced NO release was practically abolished in the presence of TTX or the specific nNOS inhibitor 7-NI, indicating a neuronal origin for this NO.

Abnormalities in endothelial NO release have been extensively validated in a range of different diabetes models and diabetic patients [3, 25]. However, there are few data regarding the effect of diabetes on neuronal NO release and its implications for vascular function. In the present study, diabetes during four weeks led to diminished perivascular neuronal NO release, which was accompanied by an enhanced contractile response elicited by nerve stimulation of rat mesenteric arteries. This augmented vasoconstriction seems to be due in part to a dysfunctional nitrergic system, as indicated by the inability of L-NAME to increase EFS-induced contractions in these arteries. In previous works using this experimental approach, we have demonstrate that the intake of a high fat diet induced hyperglycemia [26], and was associated with a decreased neuronal NO release [21]. Taken into account these results we consider that the hyperglycemic effect of streptozotocin in this study is the responsible of the diminished NO release we observed at 4 weeks.

The essential cofactor tetrahydrobiopterin ((6R)-5,6,7,8-tetrahydro-l-biopterin, BH4) plays a key role in the mechanism of NO synthesis, and a deficiency in this cofactor has been associated with many diabetic complications [27]. Limited BH4 availability not only impairs the production of NO but also, because of NOS uncoupling, leads to increased superoxide anion generation, which can also enhance BH4 oxidation and possibly help explain the limited BH4 availability [28]. Indeed, BH4 oxidation, rather than decreased BH4, seems to be the main determinant for NOS uncoupling in hyperglycaemic conditions [29]. Furthermore, the superoxide radical reacts with NO to produce the strongly oxidizing and presumably deleterious peroxynitrite radical (ONOO^–^). Endothelial NOS uncoupling and impaired endothelial NO-mediated vasodilation has been exhaustively demonstrated in diabetes [27]; however, there is limited information on the uncoupled nNOS to cause neural and vascular dysfunction. Results obtained here reveal that despite increased superoxide dismutase activity, superoxide anion generation was increased in four-week diabetic arteries. This result led us to hypothesise a possible excessive oxidation and depletion of BH4 in early diabetes, leading to nNOS uncoupling and a decrease in NO release. Indeed, our results suggest that decreased NO production in these rats may be due to uncoupled nNOS. The fact that addition of NOS co-factor, BH4, increased NO release in arteries from four-week diabetic rats provides a rational support for this conclusion.

Increased ROS generation not only induces oxidation of the NOS cofactor BH4, but also oxidises the arginine transporter, decreasing L-arginine transport and function [30, 31]. Limited L-arginine availability also results in NOS uncoupling and reduced NO release [27]. In addition, the plasma concentration and vascular content of L-arginine are reduced in diabetic animals [27, 32]. In the current study, the enhancing effect of L-arginine in the decreased neuronal NO release is consistent with deficiency in L-arginine in mesenteric segments from four-week diabetic rats. Our results seem to indicate that, as occurs in endothelial cells [32], diabetes also decreases the availability of the subtract L-arginine to NO synthesis in perivascular neurons. Moreover, the further increase in NO release observed in diabetic arteries co-incubated with L-arginine plus BH4 indicates the coexistence of two additive mechanisms.

An L-arginine deficit has been related to increased arginase activity and/or overexpression in some cardiovascular disturbances, and this alteration has emerged as an important regulator of NO release throughout the cardiovascular system [33]. In this sense, up-regulation of this metalloprotease, which competes with NOS for the common substrate L-arginine, has been described in diabetes to produce impaired NO production, increased ROS generation and endothelial dysfunction [34, 35]. Arginase exists as two distinct isoforms, arginase I and II [33]. Results obtained here indicate the coexistence of the two arginase isoforms in rat mesenteric arteries and identified arginase I overexpression as one of the possible mechanisms that participate in the decreased neuronal NO release in four-week diabetic rats. Increased arginase protein expression has also been described in cavernous tissue from diabetic rats, this observation being accompanied by decreases in nitrergic nerve relaxation responses [36].

Another mechanism that determines or influences neuronal NO release is nNOS phosphorylation. This process is regulated by various kinases and phosphatases, such as PKA, PKC, calmodulin-dependent protein kinase II (CaMKII) and phosphatase 1. Phosphorylation of nNOS at Ser^847^ reduces its activity by inhibiting Ca^2+^-CaM binding while phosphorylation at Ser^1412^ increases nNOS activity [37, 38]. Our results indicate that in arteries from four-week diabetic rats p-NOS at Ser^1417^ expression remained unmodified meanwhile p-NOS at Ser^847^ was increased. This result indicates that in early stages of diabetes the nNOS activity is decreased, impairing perivascular neuronal NO release. Furthermore, it has been recently reported that phosphorylation of nNOS at Ser^847^ also increases its uncoupling reaction leading not only to decreases in NO production but also to increased ROS generation [39], in turn producing an additive inhibitory effect on neuronal NO release. Despite this, decreased neuronal NO release in the early stages of diabetes may prevent irreversible oxidation of cellular components by limiting peroxynitrite formation from NO and superoxide anions.

As mentioned before, diabetes courses with different effects on NO release, some of these differences are time-dependent. Normal or decreased nitrergic function has been reported in diabetes [8, 12–15]. In a previous study, we suggested that eight weeks diabetes in rats courses with increased neuronal NO release in rat mesenteric arteries. This conclusion was based on the greater effect of L-NAME on EFS-induced contraction in eight-week diabetic arteries [40]. The present study extends our previous findings by examining the effect of diabetes on neuronal NO release rat in mesenteric arteries. Results obtained here show that, although there is a reduction in neuronal NO release and function four weeks after diabetes induction, on the eighth week release was restored towards levels observed in non-diabetic rats. These results indicate a time-dependent pattern of perivascular neuronal NO release in diabetes. Still, based on these results, we cannot explain this greater effect of L-NAME on EFS-induced contraction as a result of increased neuronal NO release in arteries of diabetic rats. A possible explanation for this greater L-NAME effect in diabetic arteries is its ability to blockade, in addition to neuronal NO, the counter regulatory effect of peroxynitrite on EFS-induced contraction. As we have previously reported, peroxynitrite has a vasodilator effect in rat mesenteric vascular bed [23]. Moreover, its generation is increased in eight-week diabetic arteries with respect to levels in control rats.

Taking into account the restoration of neuronal NO release, our next objective was to analyse the possible participation of the mechanisms that we have identified as the causes of changes in neuronal NO release in four-week diabetic rats. First, we have observed that superoxide anion generation remained increased in diabetic arteries, allowing us to hypothesise a BH4 deficit. However, the addition of BH4 plus L-arginine or BH4 given alone did not alter neuronal NO release in diabetic arteries. This seems to indicate nNOS recoupling. In addition, we observed a standardization of arginase I expression and, possibly, L-arginine levels as well as normalization of p-nNOS Ser^847^ expression, which could be implicated in the recovery of NO release capacity. The fact that the same levels of hyperglycemia are present at both at 4 and 8 weeks after streptozotocin treatment, suggest the existence of other mechanisms or adaptation of the cardiovascular system to hyperglycemia which could explain the transient reduction in NO release and increase in arginase 1 expression. We are conscious that in this condition where oxidative stress is increased, recovery of only these two parameters does not fully explain the restoration of the capacity to release NO, and further studies will be required to clarify these underlying mechanisms. In this sense, recent results report the development of complex mechanisms under conditions of severe oxidative stress that allow nNOS to continue to generate NO [41]. On the other hand, this recovered NO release in parallel with the increased superoxide anion generation raise peroxynitrite formation, and this could participate in the neurodegeneration widely described in chronic diabetes [42]. It is important to remark that the fact that the decreased peroxynitrite formation observed 4 weeks after treatment with streptozotocin has recovered at 8 weeks indicates the existence of transient protective mechanisms that could be relevant to delaying the development of deleterious effect in diabetes.

Our results are consistent with the existence of time-dependent effects of diabetes on neuronal NO release in rat mesenteric arteries. In the early phase, diabetes induced increased superoxide anion generation, nNOS uncoupling and overexpression of arginase I and p-nNOS Ser^847^, which resulted in decreased neuronal NO release. In a late phase, probably due to an adaptive mechanism, neuronal NO release was restored, which involves normalisation of arginase I and p-nNOS Ser^847^ expression, suggesting a nNOS recoupling.

## Abbreviations

DAF-2: 4, 5- diaminofluorescein
4W: 4 weeks
8W: 8 weeks
Arg: Arginase
DEA-NO: Diethylamine Nonoate Diammonium Salt
EFS: Electrical Field Stimulation
NA: Noradrenaline
NO: Nitric Oxide
ONOO^-^: Peroxinitrite
p-nNOS: Phosphorilated nNOS
SMA: Superior Mesenteric Artery
O_2_^.-^: Superoxide Anions
SOD: Superoxide dismutase
BH4: Tetrahydrobiopterine
TTX: Tetrodotoxin
